# *Amphibambusa fangchenggangensis* sp. nov., *Am. yunnanensis* sp. nov., and *Arecophila viscosa* sp. nov. (*Xylariales*, *Cainiaceae*) associated with bamboo from southwest China

**DOI:** 10.3897/mycokeys.132.192832

**Published:** 2026-05-22

**Authors:** Zuquan Yao, Shilian Zheng, Qing Yang, Shiping Zou, Kamran Habib, Dong-Qin Dai, Hind A. Al-Shwaiman, Abdallah M. Elgorban, Faten Zubair Filimban, Nalin N. Wijayawardene, Qirui Li

**Affiliations:** 1 State Key Laboratory of Functions and Applications of Medicinal Plants & Guizhou Key Laboratory of Microbio and Infectious Disease Prevention & Control, Guizhou Medical University, Guiyang, Guizhou, 561113, China The Institute of Biotechnology and Genetic Engineering, Chulalongkorn University Bangkok Thailand; 2 The High Efficacy Application of Natural Medicinal Resources Engineering Centre of Guizhou Province (The Key Laboratory of Optimal Utilization of Natural Medicine Resources), School of Pharmaceutical Sciences, Guizhou Medical University, Guiyang, Guizhou, 561113, China Key Laboratory of Yunnan Provincial Department of Education of the Deep-Time Evolution on Biodiversity from the Origin of the Pearl River, Qujing Normal University Qujing China; 3 Department of Entomology and Plant Pathology, Faculty of Agriculture, Chiang Mai University, Chiang Mai 50200, Thailand College of Science, King Saud University Riyadh Saudi Arabia; 4 Key Laboratory of Yunnan Provincial Department of Education of the Deep-Time Evolution on Biodiversity from the Origin of the Pearl River, Qujing Normal University, Qujing, Yunnan Province, 655011, China Center of Excellence in Biotechnology Research (CEBR), King Saud University Riyadh Saudi Arabia; 5 Department of Botany and Microbiology, College of Science, King Saud University, P.O. 2455, Riyadh 11451, Saudi Arabia Faculty of Sciences, King Abdulaziz University Jeddah Saudi Arabia; 6 Center of Excellence in Biotechnology Research (CEBR), King Saud University, Riyadh, Saudi Arabia State Key Laboratory of Functions and Applications of Medicinal Plants & Guizhou Key Laboratory of Microbio and Infectious Disease Prevention & Control, Guizhou Medical University Guiyang China; 7 Division of Plant Sciences, Department of Biological Sciences, Faculty of Sciences, King Abdulaziz University, Jeddah, Saudi Arabia School of Pharmaceutical Sciences, Guizhou Medical University Guiyang China; 8 High-Value Food from Mushrooms and Bioactive Plants in the Green Economy Value Chain Research Group, The Institute of Biotechnology and Genetic Engineering, Chulalongkorn University, 254 Phayathai Road, Pathumwan, Bangkok, 10330, Thailand Faculty of Agriculture, Chiang Mai University Chiang Mai Thailand

**Keywords:** Ascomycetous fungi, karst ecosystem, microfungi, systematics, three new species

## Abstract

During an ongoing investigation of bambusicolous fungi within the *Xylariomycetidae*, three microfungal specimens belonging to the family *Cainiaceae* were collected from southern China. Based on detailed morphological observations and phylogenetic analyses of combined internal transcribed spacer (ITS) and large subunit ribosomal RNA (LSU) sequence data, these collections were identified as two new species of *Amphibambusa*, namely *Amphibambusa fangchenggangensis* and *Am. yunnanensis*, and one new species of *Arecophila*, *Ar. viscosa*. Comprehensive descriptions, micrographs, and a phylogenetic tree of the new species are provided, highlighting bamboo forests as a rich reservoir of fungal diversity.

## Introduction

The family *Cainiaceae* represents a well-defined lineage within the order *Xylariales* (Samarakoon et al. 2022; Habib et al. 2025). Members of this family are characterized by asci with a distinctive complex apical ring and ascospores typically bearing longitudinal germ slits (Samarakoon et al. 2016; Hyde et al. 2020). Species of *Cainiaceae* are primarily saprobic or weakly parasitic on monocotyledons, including grasses, bamboos, and palms (Krug 1978; Habib et al. 2025; Ren et al. 2025).

*Arecophila* is one of the largest genera in *Cainiaceae*. It was established by Hyde (1996) to accommodate species occurring on monocotyledons and possessing striate or verrucose, brown ascospores, with *Ar. gulubiiicola* designated as the type species. The genus is characterized by immersed, subglobose to lenticular ascomata; a poorly developed or absent clypeus; and asci with a wedge-shaped apical ring that is J+ in Melzer’s reagent and two-celled, brown ascospores with wall striations that are surrounded by a mucilaginous sheath (Hyde 1996; Kang et al. 1999; Umali et al. 1999). Hyde (1996) initially placed the genus in *Amphisphaeriaceae* based on its unitunicate asci with a J+ apical ring and brown, two-celled ascospores. Kang et al. (1999) later re-evaluated the genus and transferred it to *Cainiaceae*. Subsequent molecular phylogenetic studies incorporating large subunit ribosomal RNA (LSU) and small subunit ribosomal RNA (SSU) sequence data demonstrated that *Arecophila* clusters with *Cainia* within *Xylariales* (Smith et al. 2003). Its placement in *Cainiaceae* has since been consistently supported by internal transcribed spacer (ITS)-based and multi-locus phylogenetic analyses (Jeewon et al. 2003; Senanayake et al. 2015; Hyde et al. 2020; Wijayawardene et al. 2020).

The genus *Amphibambusa* was introduced by Liu et al. (2015), with *Am. bambusicola* as the type species. Similar to *Arecophila*, it was initially placed in *Amphisphaeriaceae*, but later studies transferred it to *Cainiaceae* based on multi-locus phylogenetic evidence of ITS and LSU sequence data (Senanayake et al. 2015; Hyde et al. 2020). Species of *Amphibambusa* are predominantly reported from bamboo and are mainly distributed in tropical and subtropical regions of Asia. Many records originated from southern China, particularly Yunnan, Guizhou, and Guangxi provinces, suggesting a distinct geographical occurrence for this genus (Zhou et al. 2024; Liu et al. 2025).

During an ongoing investigation of bambusicolous fungi within *Xylariomycetidae*, several specimens belonging to *Cainiaceae* were collected from bamboo in southern China. Morphological examination and phylogenetic analyses based on ITS and LSU sequence data revealed that these specimens represent three species new to science, belonging to the genera *Amphibambusa* and *Arecophila*. Detailed descriptions, illustrations, and phylogenetic analyses of these new taxa are provided in this study.

## Materials and methods

### Sample collection and preservation

Samples were collected during field surveys conducted at Mount Foding Nature Reserve in Guizhou Province, Mount Wuliang Nature Reserve in Yunnan Province, and the Shiwandashan National Nature Reserve in the Guangxi Zhuang Autonomous Region, China, from July 2024 to September 2025. Detailed habitat information, including geographical coordinates (latitude, longitude, and altitude), was recorded during field collection. Samples were placed in sealed bags and transported to the laboratory for subsequent morphological examination and isolation of fungal strains. To preserve freshness, specimens were dried using a portable fan dryer. The dried specimens were carefully labeled and stored for further study. Subsequently, the specimens were used for morphological and molecular studies. All specimens were deposited in the Herbarium of Guizhou Medical University (**GMB**) and the Herbarium of Cryptogams, Kunming Institute of Botany, Chinese Academy of Sciences (**KUN-HKAS**).

### Morphological characterization and isolation

Macroscopic features, e.g., ostiole and clypeus, of the specimens were examined using an Olympus SZ61 stereomicroscope and photographed with a Canon 700D digital camera. Microscopic morphological characteristics, e.g., ascomata, peridium, paraphyses, asci, and ascospores, were observed using an optical microscope (Nikon Ni) and photographed with an attached Canon 700D digital camera. The amyloid reaction of the apical apparatus was tested using Melzer’s iodine reagent. The dimensions of asci and ascospores were measured using Tarosoft Image Framework (v.0.9.0.7). Images were processed using Adobe Photoshop CS6 (Adobe Systems, USA).

### DNA extraction, PCR amplification, and sequencing

Mycelium was scraped from pure culture plates using a sterilized scalpel, and genomic DNA was extracted according to the manufacturer’s instructions using the BIOMIGA Fungus Genomic DNA Extraction Kit. For specimens in which ascospores failed to germinate, DNA was extracted directly from perithecial contents. DNA samples were stored at –20 °C. The ITS region and the LSU gene were amplified by polymerase chain reaction (PCR) using primers ITS1/ITS4 (White 1990; Gardes and Bruns 1993) and LR0R/LR5 (Vilgalys and Hester 1990), respectively. The PCR reaction mixture had a total volume of 25 μL, containing 9.5 μL double-distilled water, 12.5 μL PCR Master Mix, 1 μL of each forward and reverse primer, and 1 μL template DNA. PCR products were verified by electrophoresis on a 1.5% agarose gel stained with Golden View and subsequently sent to Sangon Biotech Co., Ltd. (Shanghai, China) for sequencing. Isolation attempts were performed using the single-ascospore isolation method described by Long et al. (2019); however, these attempts were unsuccessful, and no pure cultures were obtained.

### Sequence alignment and phylogenetic analyses

All obtained sequences were deposited in GenBank (Table 1). Sequences were compared both pairwise and against available sequences in GenBank using the BLASTn algorithm for accurate identification. Molecular phylogeny was inferred from a combined dataset of ITS and LSU sequences. Reference sequences retrieved from public databases were selected based on recently published literature and BLASTn results showing high sequence similarity. Sequence alignments were performed using the MAFFT v.7.110 online program (https://mafft.cbrc.jp/alignment/server/index.html; Katoh et al. 2019) with default parameters and were manually adjusted where necessary using BioEdit v.7.0.5.3 (Hall 1999). Maximum likelihood (ML) analysis was conducted using RAxML v.8.2.12 under the GTR+G+I substitution model with 1,000 bootstrap (BS) replicates (Stamatakis 2014). Bayesian inference (BI) phylogenetic analysis was performed using MrBayes v.3.2.1 (Ronquist et al. 2012). Posterior probabilities (PP) were estimated using Markov chain Monte Carlo (MCMC) sampling in MrBayes v.3.2.2 (Ronquist et al. 2012). Six simultaneous Markov chains were run for 1,000,000 generations, with trees sampled every 1,000^th^ generation. The resulting phylogenetic tree was visualized using FigTree v.1.4.4 (Rambaut 2018). All phylogenetic analyses were conducted on the CIPRES Science Gateway v.3.3 web portal (Miller et al. 2010).

**Table 1.** Taxa and corresponding GenBank accession numbers of sequences used in the phylogenetic analysis.

Species	Strain	GenBank accession numbers		References
		ITS	LSU	
*Amphibambusa aureae*	GMB4550^T^	PQ066508	PQ066514	Zhou et al. (2024)
*Am. aureae*	GMB4561	PQ066509	PQ066515	Zhou et al. (2024)
*Am. aquatica*	MFLUCC 18-1046^T^	PP584660	PP584659	Manawasinghe et al. (2025)
*Am. bambusicola*	MFLU 14-0825^T^	KP744433	KP744474	Liu et al. (2015)
*Am. bambusicola*	GMB5602^T^	PQ884689	PQ885401	Liu et al. (2025)
*Am. bambusicola*	GMB5608	PQ884690	PQ885402	Liu et al. (2025)
*Am. cerosissimae*	GMB5603^T^	PQ884693	PQ885405	Liu et al. (2025)
*Am. cerosissimae*	GMB5613	PQ884694	PQ885406	Liu et al. (2025)
*Am. guangxiensis*	GMB4910^T^	PV617366	PV617364	Yao et al. (2025)
*Am. guangxiensis*	GMB4911	PV617365	PV617363	Yao et al. (2025)
*Am. hongheensis*	KUN-HKAS112723^T^	MW892971	MW892969	Jiang et al. (2021)
*Am. hongheensis*	KUMCC 20-0334	MW892972	MW892970	Jiang et al. (2021)
*Am. subbambusicola*	GMB5606^T^	PQ884691	PQ885403	Liu et al. (2025)
*Am. subbambusicola*	GMB5618	PQ884692	PQ885404	Liu et al. (2025)
*Am. xishuangbannaensis*	GMB5601^T^	PV644573	PV644575	Ren et al. (2025)
*Am. xishuangbannaensis*	GMB5634	PV644574	PV644576	Ren et al. (2025)
*Am. fangchenggangensis*	GMB7602^T^	PX932424	PZ106884	This Study
*Am. fangchenggangensis*	GMB7702	PX932425	PZ106885	This Study
*Am. yunnanensis*	GMB7603^T^	PX932422	PZ106882	This Study
*Am. yunnanensis*	GMB7703	PX932423	PZ106883	This Study
*Arecophila amphibambusina*	GMB6259^T^	PQ874066	PQ860514	Habib et al. (2025)
*Ar. amphibambusina*	GMB6260	PQ874067	PQ860515	Habib et al. (2025)
*Ar. australis*	GZUCC0124	MT742125	MT742132	Li et al. (2022)
*Ar. australis*	GZUCC0112^T^	MT742126	MT742133	Li et al. (2022)
*Ar. bambusae*	HKUCC 4794	N/A	AF452038	Umali et al. (1999)
*Ar. chinensis*	GMB6217^T^	PQ874007	PQ860452	Habib et al. (2025)
*Ar. chinensis*	GMB6218	PQ874008	PQ860453	Habib et al. (2025)
*Ar. clypeata*	GZUCC0127	MT742128	MT742135	Li et al. (2022)
*Ar. clypeata*	GZUCC0110^T^	MT742129	MT742136	Li et al. (2022)
*Ar. clypeata*	MFLU 18-2280	OR224995	OP612558	Zhang et al. (2023)
*Ar. gaofengensis*	GMB4541^T^	PQ066512	PQ066516	Zhou et al. (2024)
*Ar. gaofengensis*	GMB4559	PQ066513	PQ066517	Zhou et al. (2024)
*Ar. guizhouensis*	GMB1138^T^	PQ884695	PQ885407	Liu et al. (2025)
*Ar. guizhouensis*	GMB5614	PQ884696	PQ885408	Liu et al. (2025)
*Ar. miscanthi*	MFLU 19-2333^T^	NR171235	NG088086	Hyde et al. (2020)
*Ar. miscanthi*	FU31025	MK503821	MK503827	Hyde et al. (2020)
*Ar. miscanthi*	GZAAS 19-1753	OR224996	OP612557	Zhang et al. (2023)
*Ar. maolanensis*	HGUP24-0024^T^	PQ568137	PQ569318	Sun et al. (2025)
*Ar. muroiana*	GZUCC0122	MT742127	MT742134	Li et al. (2022)
*Ar. subguizhouensis*	GMB5617^T^	PQ884697	PQ885409	Liu et al. (2025)
*Ar. subguizhouensis*	GMB5610	PQ884698	PQ885410	Liu et al. (2025)
*Ar. xishuangbannaensis*	GMB-W1283^T^	OR995736	OR995743	Han et al. (2024)
*Ar. xishuangbannaensis*	ZHKU23-0280	OR995737	OR995744	Han et al. (2024)
*Ar. yunnanensis*	HKAS 136906	N/A	PQ569319	Sun et al. (2025)
*Ar. zhaotongensis*	GMBCC1145^T^	OR995740	OR995747	Han et al. (2024)
*Ar. zhaotongensis*	ZHKU23-0260	OR995738	OR995745	Han et al. (2024)
*Ar.* sp.	HKUCC 6487	N/A	AF452039	Jeewon et al. (2003)
*Ar. viscosa*	GMB7601^T^	PX932426	PZ106886	This Study
*Ar. viscosa*	GMB7701	PX932427	PZ106887	This Study
*Atrotorquata lineata*	Mt25	AF009807	N/A	Unpublished
*Barrmaelia moravica*	CBS 142769^T^	NR153495	N/A	Voglmayr et al. (2018)
*B. rhamnicola*	CBS 142772^T^	NR153497	N/A	Voglmayr et al. (2018)
*B. rappazii*	CBS 142771^T^	NR153496	N/A	Voglmayr et al. (2018)
*Cainia anthoxanthis*	MFLUCC 15-0539^T^	NR138407	NG070382	Senanayake et al. (2015)
*C. desmazieri*	CBS 137.62	MH858124	MH869702	Vu et al. (2019)
*C. daweishanensis*	GMB5619^T^	PQ884699	PQ885411	Liu et al. (2025)
*C. daweishanensis*	GMB5624	PQ884700	PQ885412	Liu et al. (2025)
*C. globosa*	MFLUCC 13-0663^T^	NR171724	KX822123	Unpublished
*C. graminis*	CBS 136.62	MH858123	MH869701	Vu et al. (2019)
*C. graminis*	LSU0203	MT000398	MT000493	Unpublished
*C. shilihetanensis*	GMB5612^T^	PQ884701	PQ885413	Liu et al. (2025)
*C. shilihetanensis*	GMB5622	PQ884702	PQ885414	Liu et al. (2025)
*Endocalyx cinctus*	JCM 7946	LC228648	LC228704	Unpublished
*E. cinctus*	CC-HSC6	PP407858	PP407674	Unpublished
*E. cinctus*	NBRC 31306	MZ313191	MZ313152	Unpublished
*E. metroxyli*	MFLUCC 15-0723B	MT929163	MT929314	Konta et al. (2021)
*E. metroxyli*	MFLUCC 15-0723A^T^	NR176745	NG081486	Konta et al. (2021)
*E. metroxyli*	MFLUCC 15-0723C	N/A	MT929315	Konta et al. (2021)
*E. ptychospermatis*	UESTCC:23.0130	OR253165	N/A	Unpublished
*E. ptychospermatis*	SNT334	PP592386	N/A	Zhang et al. (2024)
*E. indumentum*	JCM 8042	N/A	MZ313157	Gregorio et al. (2021)
*E. indumentum*	JCM 5171	N/A	MZ313161	Gregorio et al. (2021)
*E. phoenicis*	ZHKUCC 22-0128	OR164915	N/A	Senanayake et al. (2023)
*E. phoenicis*	ZHKUCC 22-0135	OR164914	N/A	Senanayake et al. (2023)
*Longiappendispora chromolaenae*	MFLUCC 17-1485^T^	NR169723	NG068714	Mapook et al. (2020)

Notes: Type specimens are marked with T; “N/A” indicates that no sequence is available in GenBank; newly generated sequences are indicated in red.

## Results

### Phylogeny

**Figure 1.**
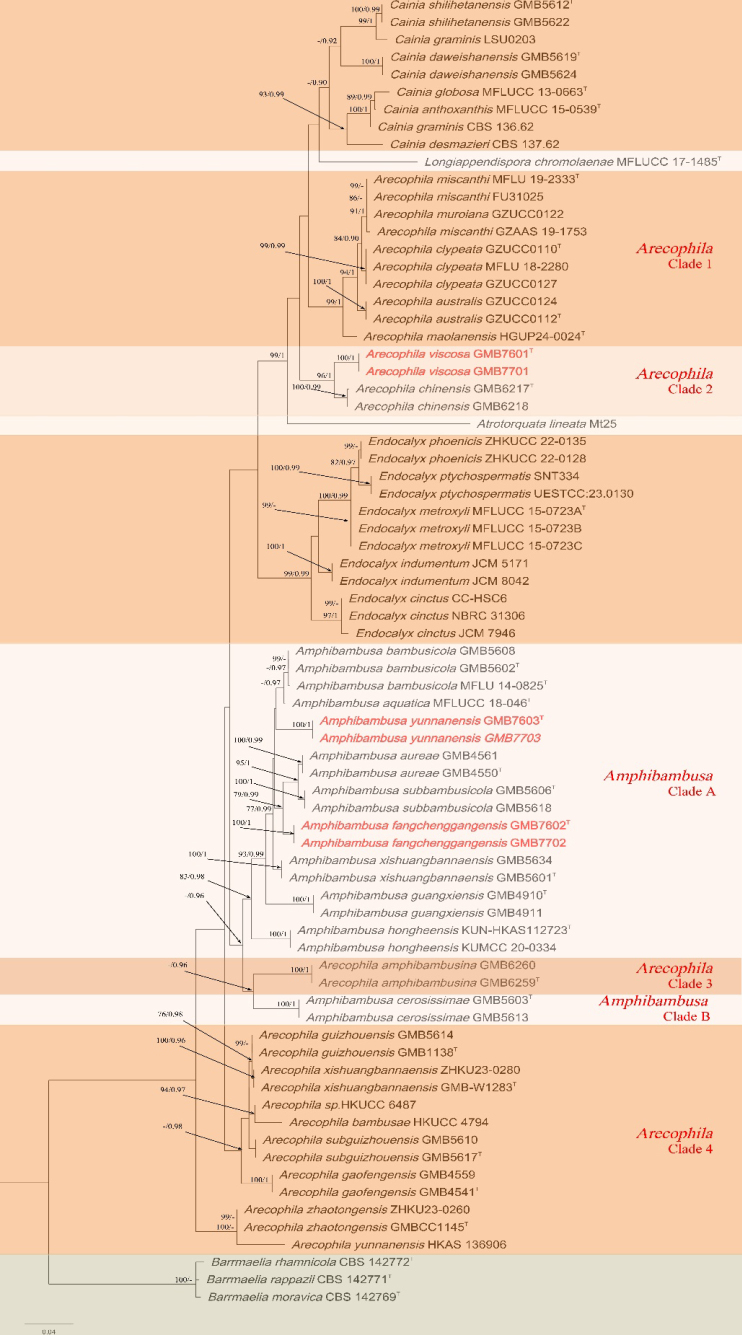
Phylogenetic tree of *Arecophila* and *Amphibambusa* with related taxa based on combined ITS and LSU sequences. Nodes with bootstrap support values ≥ 70% (ML) and posterior probabilities (PP) ≥ 0.90 are indicated (ML/PP). Type materials are marked with “T,” and newly described species are highlighted in red.

After excluding ambiguously aligned regions and long gaps, the final concatenated dataset comprised 1,400 characters (ITS: 1–517, LSU: 518–1400). Three *Barrmaelia* species, viz., *B. moravica* (CBS 142769), *B. rhamnicola* (CBS 142772), and *B. rappazii* (CBS 142771), were used as outgroup taxa. The phylogenetic analyses based on ML and BI produced highly congruent tree topologies. The best-scoring ML tree is shown in Fig. 1, with a final optimization likelihood value of -8071.573220. The estimated parameters of the GTR+G+I model were as follows: base frequencies A = 0.251, C = 0.234, G = 0.282, T = 0.233; substitution rates A↔C = 2.010, A↔G = 3.796, A↔T = 2.812, C↔G = 1.717, C↔T = 8.502, and G↔T = 1.000. The gamma distribution shape parameter (*α*) was 0.540, and the tree length was 1.567.

In the phylogram (Fig. 1), *Amphibambusa yunnanensis* (GMB7603, GMB7703) formed a clade sister to *Am. aquatica* and *Am. bambusicola*. Although this relationship received low bootstrap support, the clade remained consistently stable across repeated phylogenetic analyses. The sequences of *Am. fangchenggangensis* (GMB7602, GMB7702) formed a well-supported (91% BS/1.0 PP) independent lineage sister to the clade comprising *Am. aureae* and *Am. subbambusicola*, supporting its recognition as a distinct species. Within the genus *Arecophila*, *Ar. viscosa* (GMB7601, GMB7701) formed a distinct clade with *Ar. chinensis* as its sister species.

### Taxonomy

#### Amphibambusa fangchenggangensis

Taxon classificationFungiAmphisphaerialesAmphisphaeriaceae

Z.Q. Yao & Q. R. Li sp. nov.

9077C599-6F4E-5865-9C6C-493103010EBA

862737

Fig. 2

##### Etymology.

The specific epithet ‘*fangchenggangensis*’ refers to the locality of the type collection, Fangchenggang City, Guangxi Zhuang Autonomous Region, China.

##### Holotype.

China • Guangxi Zhuang Autonomous Region, Fangchenggang City, Shiwangdashan National Nature Reserve (21°42'93.19"N, 107°19'16.67"E), altitude 754 m, on dead bamboo culms, 14 August 2024, Z.Q. Yao, 2024SWS100 (GMB7602, holotype); *ibid*. KUN-HKAS 152927, isotype.

##### Description.

***Saprobic*** on decaying bamboo culms, forming black, rounded spots on the host surface. ***Ascomata*** 547–875 × 524–817 µm (x̄ = 728.4 × 676.8 µm, *n* = 15), immersed, solitary to scattered, globose to oval-subglobose, black, ostiolate. ***Ostiole*** 73.9–169.2 × 117.4–170.8 µm (x̄ = 126.6 × 146.9 µm, *n* = 10), centric, periphysate. ***Peridium*** 13–28 µm (x̄ = 22.5 µm, *n* = 10) wide, composed of thick-walled, hyaline to brown cells, ***textura angularis***. ***Paraphyses*** 2.5–9.5 µm (x̄ = 4.1 µm) wide, filamentous, aseptate, thin-walled, with hyaline, guttulate cells. ***Asci*** 211–274 × 12–23 µm (x̄ = 238.5 × 15.5 µm, *n* = 25), 8-spored, unitunicate, cylindrical, short-stalked, with a J+, disc-shaped, subapical ring, blues in Melzer’s reagent, measuring 3–5 × 2–4 µm (x̄ = 4.5 × 2.5 µm, *n* = 10). ***Ascospores*** 23.5–35.5 × 6–10 µm (x̄ = 30.9 × 7.9 µm, *n* = 30), uniseriate, hyaline, fusiform, verrucose, 1-septate, slightly constricted at the septum, tapering towards both ends, with longitudinal striations along their entire length, surrounded by a mucilaginous sheath 9.5–15 µm (x̄ = 12.2 µm, *n* = 10) thick. **Asexual morph**: Undetermined.

**Figure 2.**
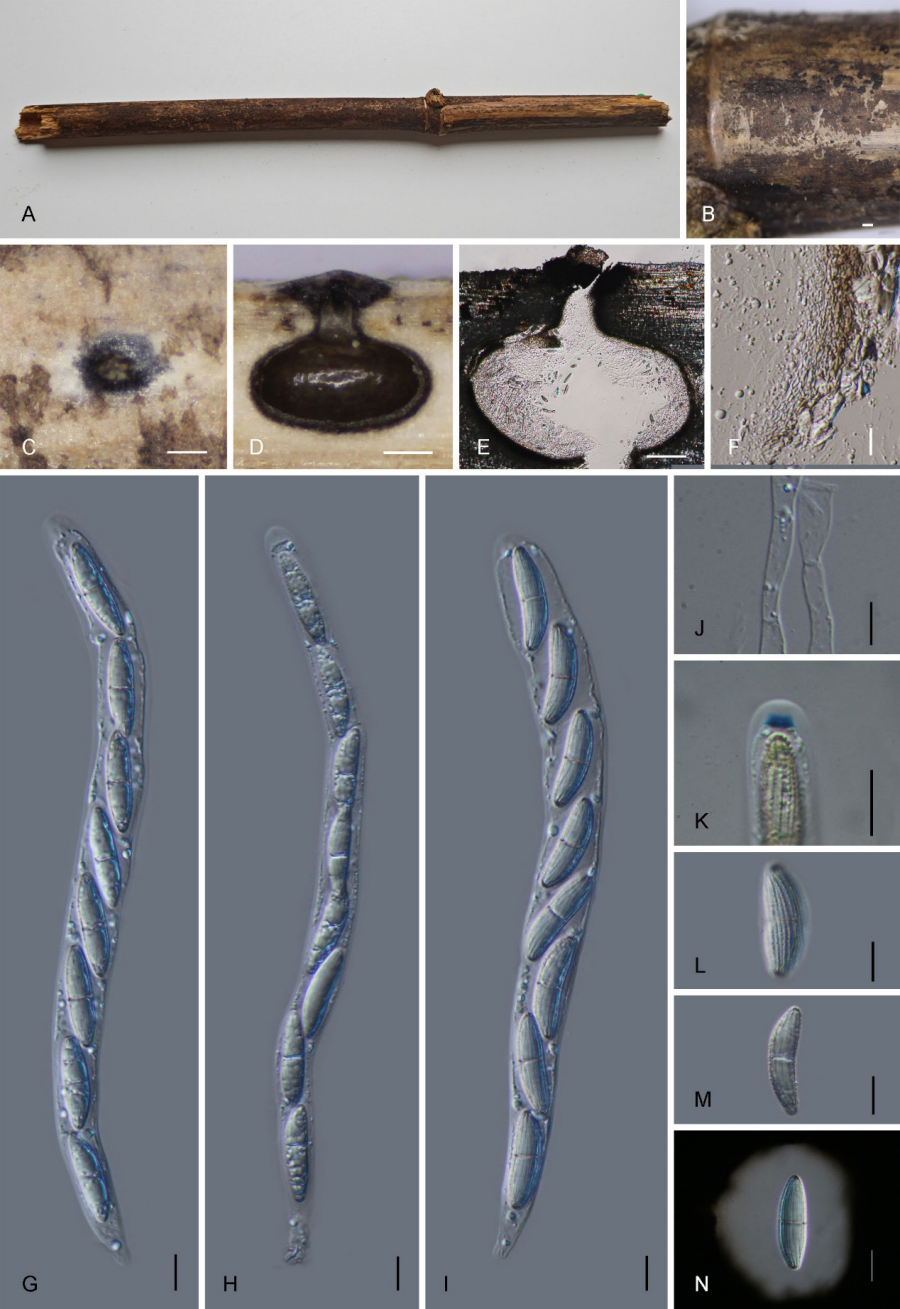
*Amphibambusa fangchenggangensis* (GMB7602, holotype). **A**. Bamboo host; **B, C**. Ascomata on the surface of the host; **D, E**. Longitudinal sections of ascomata; **F**. Peridium; **G–I**. Asci in distilled water; **J**. Paraphyses; **K**. A J+ apical apparatus bluing in Melzer’s reagent; **L–N**. Ascospores (**N**: in Indian ink). Scale bars: 0.2 mm (**B–D**); 100 µm (**E**); 10 µm (**F–N**).

##### Paratype.

China • Guangxi Zhuang Autonomous Region, Fangchenggang city, Shiwangdashan National Nature Reserve (21°42'93.11"N, 107°19'16.68"E), altitude 723 m, on dead bamboo, 14 August 2024, Z.Q. Yao, 2024SWS200 (GMB7702).

##### Notes.

In the phylogenetic tree, *Amphibambusa fangchenggangensis* (GMB7602) clusters with *Am. aureae* (GMB4550) and *Am. subbambusicola* (GMB5606), forming a well-supported sister clade (91% BS/1.0 PP; Fig. 1). Morphologically, *Am. fangchenggangensis* differs from *Am. aureae* by having larger asci (211–274 × 12–23 µm vs. 90–190 × 9–18 µm) and larger ascospores (23.5–35.5 × 6–10 µm vs. 15–22.5 × 5–7.9 µm) (Zhou et al. 2024; Liu et al. 2025). In addition, the ascospores of *Am. fangchenggangensis* possess distinct longitudinal striations, whereas those of *Am. subbambusicola* are smooth. The new species also has a thicker mucilaginous sheath (9.5–15 µm) compared with *Am. aureae* (2.5–7 µm) and *Am. subbambusicola* (6–10 µm) (Zhou et al. 2024; Liu et al. 2025). Comparative sequence analyses revealed that *Am. fangchenggangensis* differs from *Am. aureae* (GMB4550) and *Am. subbambusicola* (GMB5606) by 6% (26/431 bp) and 6.6% (31/468 bp) in the ITS region, respectively, and by 1% (8/842 bp) from both taxa in the LSU region. These combined morphological and molecular differences support the recognition of *Am. fangchenggangensis* as a distinct species.

#### Amphibambusa yunnanensis

Taxon classificationFungiAmphisphaerialesAmphisphaeriaceae

Z.Q. Yao & Q. R. Li sp. nov.

51DEDC75-2755-5E30-AC2B-DBAF783A25EA

862738

Fig. 3

##### Etymology.

The specific epithet ‘*yunnanensis*’ refers to Yunnan Province, China, the locality of the type collection.

##### Holotype.

China • Yunnan Province, Wuliangshan National Nature Reserve (24°25'33.76"N, 101°23'55.57"E), altitude 1467 m, on dead bamboo culms, 16 September 2025, Z.Q. Yao, 2025WLS39 (GMB7603, holotype); *ibid*. KUN-HKAS 152930, isotype.

##### Description.

***Saprobic*** on decaying bamboo culms, forming black, round spots. ***Ascomata*** 953–1340 × 760–1003 µm (x̄ = 1109 × 862 µm, *n* = 15), immersed, solitary, scattered, globose to subglobose, black, with a central ostiole. ***Peridium*** 12.5–28 µm (x̄ = 19.1 µm, *n* = 10) wide, composed of thick-walled, hyaline to brown cells of ***textura angularis***. ***Paraphyses*** 1.5–5.5 µm (x̄ = 2.9 µm) wide, filamentous, aseptate, thin-walled, with hyaline, guttulate cells. ***Asci*** 236–290 × 12.5–17.5 µm (x̄ = 269.1 × 15.5 µm, *n* = 20), 8-spored, unitunicate, cylindrical, short-stalked, with a J+ wedge-shaped apical apparatus 3–4 × 2–3.5 µm (x̄ = 3.5 × 2.5 µm, *n* = 10). ***Ascospores*** 30–40 × 6.5–10 µm (x̄ = 35.5 × 8.2 µm, *n* = 30), uniseriate, hyaline, fusiform, 1-septate at the center, slightly constricted at the septum, tapering at both ends, with longitudinal striations along their entire length, surrounded by a mucilaginous sheath 1.7–5.5 µm (x̄ = 2.5 µm. *n* = 10) thick. **Asexual morph**: Undetermined.

**Figure 3.**
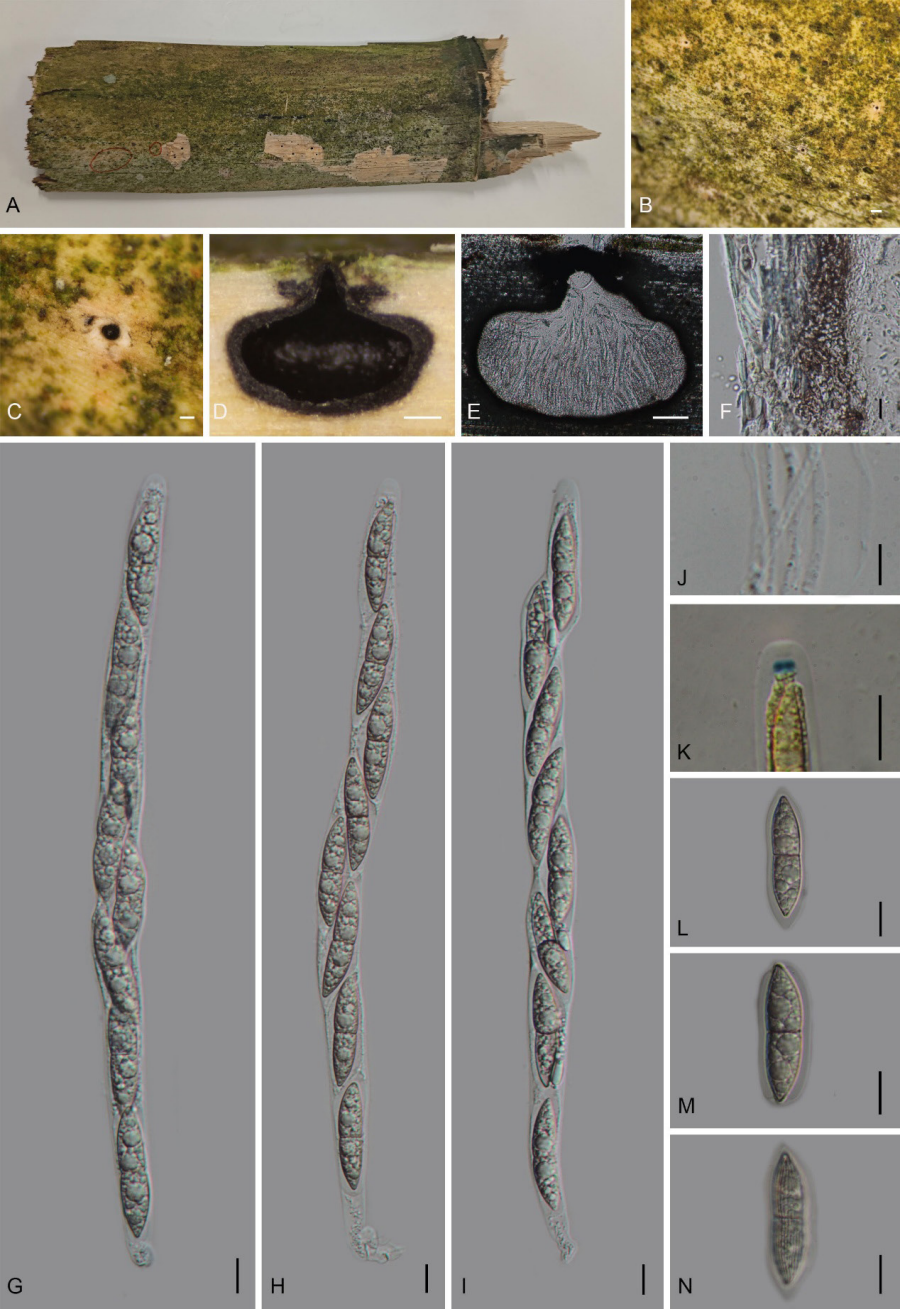
*Amphibambusa yunnanensis* (GMB7603, holotype). **A**. Bamboo host; **B, C**. Ascomata on the surface of the host; **D, E**. Longitudinal sections of ascomata; **F**. Peridium; **G–I**. Asci in distilled water; **J**. Paraphyses; **K**. A J+ apical apparatus bluing in Melzer’s reagent; **L–N**. Ascospores. Scale bars: 0.2 mm (**B–D**); 100 µm (**E**); 10 µm (**F–N**).

##### Paratype.

China • Yunnan Province, Puer city, Mount Wuliang National Nature Reserve (24°25'33.70"N, 101°23'55.52"E), altitude 1455 m, on dead bamboo culms, 16 September 2025, Z.Q. Yao, 2025WLS121 (GMB7703).

##### Notes.

In the phylogenetic tree (Fig. 1), *Amphibambusa yunnanensis* (GMB7603) is closely related to *Am. aquatica* (MFLUCC 18-1046) and *Am. bambusicola* (GMB5602). Morphologically, *Am. yunnanensis* is distinguished from *Am. aquatica* by its longer asci (236–290 µm vs. 190–240 µm) and wider ascospores (6.5–10 µm vs. 6–7.5 µm). Furthermore, *Am. aquatica* occurs on submerged decaying wood in freshwater habitats, whereas *Am. yunnanensis* was collected from dead bamboo in a terrestrial habitat (Manawasinghe et al. 2025; Liu et al. 2025). Compared to *Am. bambusicola*, the new species has larger asci (236–290 × 12.5–17.5 µm vs. 179–196 × 14.0–16.7 µm), larger ascospores (30–40 × 6.5–10 µm vs. 29.7–36.9 × 6.7–7.8 µm), and a thinner mucilaginous sheath (1.7–5.5 µm vs. up to 10 µm). Comparative sequence analysis revealed that *Am. yunnanensis* differs from *Am. aquatica* (MFLUCC 18-1046) and *Am. bambusicola* (GMB5602) by 5.4% (26/461 bp and 25/467 bp, respectively) in the ITS region, and by 1.9% (16/840 bp and 16/848 bp, respectively) in the LSU region. Based on these molecular and morphological differences, supported by phylogenetic evidence, *Am. yunnanensis* is introduced as a new species.

#### Arecophila viscosa

Taxon classificationFungiXylarialesCainiaceae

Z.Q. Yao, K. Habib & Q. R. Li sp. nov.

75C92679-36BA-5988-8D8B-F1489D8480E8

862739

Fig. 4

##### Etymology.

The specific epithet ‘*viscosa*’ refers to the viscid sheath surrounding the ascospores, a distinctive morphological characteristic of this species.

##### Holotype.

China • Guizhou Province, Fodingshan National Nature Reserve (27°19'41.16"N, 108°8'29.74"E), altitude 695 m, on dead bamboo, 16 September 2025, Z.Q. Yao, 2025FDS20 (GMB7601, ***holotype***); *ibid*. KUN-HKAS 152928, ***isotype***.

##### Description.

***Saprobic*** on dead bamboo culms. **Sexual morph**: ***Ascomata*** 506–789 × 640–795 µm (x̄ = 626 × 710 µm, *n* = 8), immersed under a black clypeus, solitary, slightly raised and dome-shaped, scattered or aggregated, subglobose to globose, with a central conical papilla visible in vertical section. ***Ostioles*** elongated, central, black, periphysate. ***Peridium*** 19–30 µm thick (x̄ = 25 µm, *n* = 10), multi-layered, outer layer composed of brown, thick-walled cells of ***textura angularis***, inner layer hyaline. ***Paraphyses*** 2.3–4.3 µm wide (x̄ = 3.2 µm, *n* = 15), hyaline, unbranched, septate. ***Asci*** 172–202 × 8.4–13.2 µm (x̄ = 185.6 × 11.2 µm, *n* = 20), 8-spored, unitunicate, long-cylindrical, with a long pedicel, apex obtuse, containing a square, J+ apical ring (2.7–3.4 × 1.6–2.4 µm, *n* = 10). ***Ascospores*** 14–19 × 5.9–7.6 µm (x̄ = 16.9 × 5.9 µm, *n* = 30), overlapping uniseriate, 2-celled, pale yellow to yellowish brown, equilateral ellipsoidal, constricted at the septum, with a longitudinal shallow groove running the entire length of the spore, surrounded by a 2.6–13.6 µm thick mucilaginous sheath, lacking germ slits and appendages. **Asexual morph**: Undetermined.

**Figure 4.**
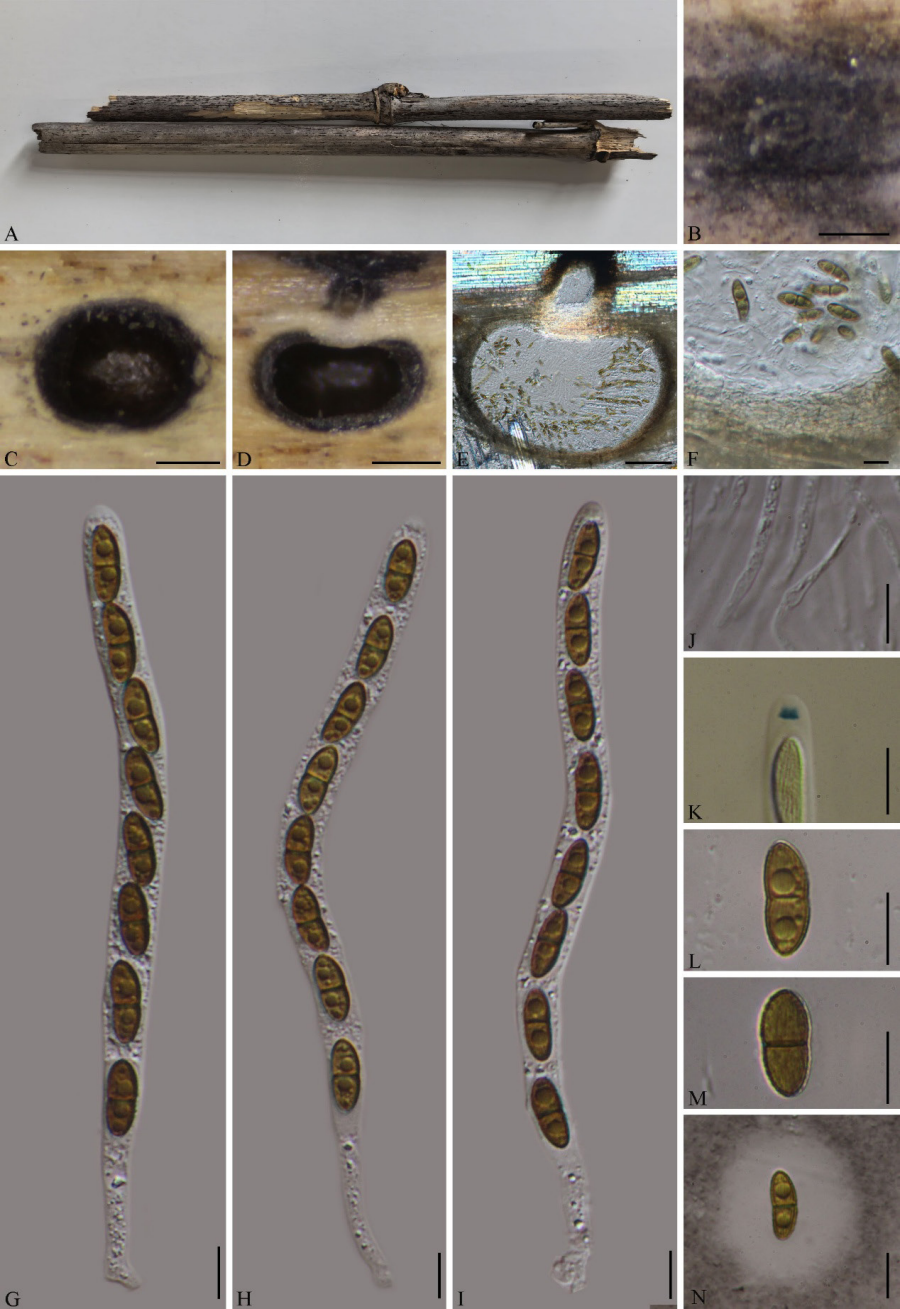
*Arecophila viscosa* (GMB7601, holotype). **A**. Bamboo host; **B, C**. Ascomata on the surface of the host; **D, E**. Longitudinal sections of ascomata; **F**. Peridium; **G–I**. Asci in distilled water; **J**. Paraphyses; **K**. A J+ apical apparatus bluing in Melzer’s reagent; **L–N**. Ascospores (**N**: in Indian ink). Scale bars: 0.2 mm (**B–D**); 100 μm (**E**); 10 µm (**F–N**).

##### Paratype.

China • Guizhou Province, Tongren city, Fodingshan National Nature Reserve (27°19'41.11"N, 108°8'29.72"E), altitude 690 m, on dead bamboo, 16 September 2025, Z.Q. Yao, 202FDS103 (GMB7701).

##### Notes.

In the phylogram (Fig. 1; *Arecophila* clade 2), *Arecophila viscosa* (GMB7601) forms a well-supported (96% BS/1.0 PP) sister clade with *Ar. chinensis* (GMB6217) and also shares morphological similarities, including immersed ascomata, an amyloid ascal apical apparatus, septate paraphyses, and brown, 1-septate ascospores. *Arecophila viscosa* is distinguished from *Ar. chinensis* by its smaller ascomata (506–789 × 640–795 µm vs. 973–1338 × 746–966 µm) and shorter asci (172–202 µm vs. 215–240 µm). Furthermore, the ascospores of *Ar. viscosa* are surrounded by a distinct, thick mucilaginous sheath (2.6–13.6 µm thick), whereas the sheath is absent in *Ar. chinensis* (Habib et al. 2025). Further, comparative nucleotide analysis revealed that *Ar. viscosa* differs from *Ar. chinensis* (GMB6217) by 1.2% (5/426 bp) in the ITS region and 0.1% (1/849 bp) in the LSU region.

Compared to *Ar. subguizhouensis* (clustered in *Arecophila* clade 4; Fig. 1), which also possesses ascospores with a sheath, *Ar. viscosa* has smaller ascospores (14.5–18.2 × 5.9–7.6 µm vs. 21–26 × 6–8.5 µm) and a slightly thicker sheath (2.6–13.6 µm vs. 6.5–9 µm). In addition, the ascospores of *Ar. viscosa* are ellipsoidal with a longitudinal shallow groove, whereas those of *Ar. subguizhouensis* are fusiform with longitudinal striations (Liu et al. 2025). Based on these molecular and morphological differences, supported by phylogenetic evidence, *Ar. viscosa* is introduced as a new species.

## Discussion

The phylogenetic position of the genus *Arecophila* reveals significant complexity (Fig. 1). Multiple studies indicate that *Arecophila* is polyphyletic, with species scattered across different clades within the family (e.g., Habib et al. 2025; Yao et al. 2025; Ren et al. 2025). Some species occupy more basal positions in the phylogeny, whereas others form clades higher up in the tree (Fig. 1). This scattered arrangement suggests that the current circumscription of the genus does not accurately reflect evolutionary relationships. Such a pattern may indicate either convergent evolution of morphological characters or the need for taxonomic reassessment.

A notable example is the description of *Arecophila amphibambusina* by Habib et al. (2025), which clusters phylogenetically within the genus *Amphibambusa* rather than with core *Arecophila* species. This placement contradicts its morphology, as the species exhibits the diagnostic features of the genus *Arecophila*, particularly brown ascospores. The discordance between molecular phylogeny and morphology raises important systematic questions, suggesting that pigmentation and other morphological traits previously considered taxonomically informative may be homoplasious and insufficient for defining generic boundaries within this group.

The basal clade of *Arecophila* species (Fig. 1; *Arecophila* clade 4) likely represents a distinct evolutionary lineage that warrants recognition as a new genus. However, these taxa morphologically conform closely to the original generic concept of *Arecophila*, which is characterized by immersed, subglobose to lenticular ascomata, asci with a wedge-shaped apical ring, and two-celled, brown ascospores with longitudinally striated walls. This discordance between phylogeny and morphology underscores the need for taxonomic revision, potentially involving the segregation of clades into separate genera while retaining or emending *Arecophila* for a more monophyletic core group.

Host association patterns provide additional insight into evolutionary relationships within the genus. Most species of *Arecophila* have been reported from bamboo hosts, suggesting a degree of host specialization that may have influenced diversification. However, three species deviate from this pattern: *Ar. australis* occurs on *Phragmites australis*, *Ar. miscanthi* on *Miscanthus sinensis*, and *Ar. clypeata* on an unidentified gramineous host. These three species form a well-supported monophyletic clade in the phylogenetic analysis. This correlation between host preference and phylogenetic grouping suggests that host shifts within *Poaceae* may have influenced lineage divergence.

The clustering of non-bamboo-associated species into a single clade may indicate an evolutionary transition from bamboo to other grasses. Alternatively, it may represent an independent lineage specialized on non-bamboo hosts. This ecological differentiation further supports the hypothesis that *Arecophila*, as currently circumscribed, comprises multiple evolutionary lineages that may deserve taxonomic recognition at the generic level.

Taken together, evidence from phylogeny, morphology, and host association strongly supports the need for a comprehensive taxonomic revision of the family. Future studies incorporating additional taxa, broader geographic sampling, multi-locus datasets, and genomic analyses will be essential to clarify generic boundaries and better understand evolutionary relationships within the family.

## Supplementary Material

XML Treatment for Amphibambusa fangchenggangensis

XML Treatment for Amphibambusa yunnanensis

XML Treatment for Arecophila viscosa

## Additional information

### Artificial Intelligence (AI) use

The authors accept full responsibility for the content of the manuscript, including the disclosure of any use of AI.

No AI tools were used in the preparation of this manuscript.

### Author ORCIDs

Z. Yao https://orcid.org/0009-0001-9434-1951

S. Zheng https://orcid.org/0009-0004-2495-4906

Q. Yang https://orcid.org/0009-0006-2794-5583

S. Zou https://orcid.org/0009-0001-8515-6885

K. Habib https://orcid.org/0000-0003-2572-0306

D.-Q. Dai https://orcid.org/0000-0001-8935-8807

A.M. Elgorban https://orcid.org/0000-0003-3664-7853

F.Z. Filimban https://orcid.org/0000-0001-7527-1313

N.N. Wijayawardene https://orcid.org/0000-0003-0522-5498

Q. Li https://orcid.org/0000-0001-8735-2890

### Data availability

All of the data that support the findings of this study are available in the main text.
